# Laser-Facilitated Additive Manufacturing Enables Fabrication of Biocompatible Neural Devices

**DOI:** 10.3390/s20226614

**Published:** 2020-11-19

**Authors:** Ailke Behrens, Jan Stieghorst, Theodor Doll, Ulrich P. Froriep

**Affiliations:** 1 Cluster of Excellence Hearing4All, 30627 Hannover, Germany; behrens.ailke@mh-hannover.de (A.B.); doll.theodor@mh-hannover.de (T.D.); 2 BioMaterial Engineering, Department of Otorhinolaryngology, Hannover Medical School, Carl Neuberg-Str. 1, 30625 Hannover, Germany; stieghorst.jan@otojig.com; 3 Fraunhofer Institute for Toxicology and Experimental Medicine ITEM, Nikolai-Fuchs-Str. 1, 30625 Hannover, Germany

**Keywords:** additive manufacturing, medical grade silicone rubber, IR curing, biocompatibility, impedance, electrophysiology, neural implant, ECoG, medical rapid prototyping

## Abstract

Current personalized treatment of neurological diseases is limited by availability of appropriate manufacturing methods suitable for long term sensors for neural electrical activities in the brain. An additive manufacturing process for polymer-based biocompatible neural sensors for chronic application towards individualized implants is here presented. To process thermal crosslinking polymers, the developed extrusion process enables, in combination with an infrared (IR)-Laser, accelerated curing directly after passing the outlet of the nozzle. As a result, no additional curing steps are necessary during the build-up. Furthermore, the minimal structure size can be achieved using the laser and, in combination with the extrusion parameters, provide structural resolutions desired. Active implant components fabricated using biocompatible materials for both conductive pathways and insulating cladding keep their biocompatible properties even after the additive manufacturing process. In addition, first characterization of the electric properties in terms of impedance towards application in neural tissues are shown. The printing toolkit developed enables processing of low-viscous, flexible polymeric thermal curing materials for fabrication of individualized neural implants.

## 1. Introduction

Despite continuous development of neural devices employing novel sensor functionalities, materials, and manufacturing processes in the last decades, only very few technologies transition from basic research to application in human patients. Thus, the current gold standards of treatment for patients suffering from neurological diseases, such as epilepsy, and loss of modalities, such as hearing or vision, are largely based on technologies developed during the second half of the last century, come in standard sizes and hence are not matching the individual patient’s anatomy [1,2]. This is likely caused by lack of manufacturing technologies, such as additive manufacturing, suitable for these applications when also considering the requirements in terms of biocompatibility of materials used. Consequently, the technology exists in principle, but it cannot be considered safe in terms of the application in medical devices for human patients. An additional caveat stems from the materials requirements imposed through neural tissue itself: ideally, neural implants should be in close contact to the neurons but unperceivable for the tissue to avoid foreign body responses commonly leading to insulation of the sensors and concomitantly to a decrease in signal-to-noise-ratio for recording [3,4]. In general, neural implants consist of a flexible electrically insulating cladding, conductive pathways for signal transmission, and the connector for the measurement set-up. There, soft materials, such as polymers, are advantageous since their mechanical properties can be fabricated to match those of the tissue [5]. Furthermore, high-quality long-term recording of neural activity depends on the electrical properties of the electrodes, primarily the impedance but also the electrode diameter for a given application. While microelectrodes with small diameters and high impedances are suitable for electrophysiological recording of activity from single neurons or small neuronal assemblies, larger diameters and lower impedances enable recording of brain oscillations reflecting population activities of larger brain networks as, e.g., used in electrocorticography (ECoG) for epilepsy diagnosis and treatment [6]. While, for the latter application, different approaches exist, optimization is desired: Especially, the flexibility of the implants (e.g., silicone rubber: young’s modulus 1 MPa [7], platinum: young’s modulus 165 GPa [8]), and the complex and time-consuming manufacturing process in clean rooms [9,10] limit advanced patient-individual processing. Taken together, a manufacturing process for neural implants reflecting the specific anatomy of individual patients requires biocompatible soft materials for cladding and electrodes that exhibit application-specific electrode impedances and sizes.

While several procedures for additive manufacturing of polymers are established, processing of flexible medical grade silicone rubber is still limited. Above all, the curing process while printing is challenging. Temperature-based, UV-initiated or humidity-activated curing are conventional curing methods of silicone rubber, while the thermal curing is the gold standard for medical applications. Especially, temperature-based and humidity-activated materials need several hours for complete curing. In comparison, it is possible to cure each layer after printing, if UV-initiated silicone rubbers are used. Extrusion or dispensing is often used for applying the silicone rubber on the printer bed, as shown, e.g., by Envisiontec GmbH (Gladbeck, Germany) or Aceo (Burghausen, Germany). Consequently, since curing is challenging, the viscosity needs to be comparably high in order to print with high shape retention capability [11,12,13]. Due to viscous materials and the processability, there are limitations for printing in small dimensions. If the viscosity is too high for the used nozzle, clogging is a prevalent challenge. In contrast to that, Udofia et al. reached very small structure sizes with a low viscosity silicone rubber in the range of 10 µm by using a nozzle with an inner diameter of 10 µm [14]. However, printing times in this dimension are extremely long and the structure size is only useful for a limited range of applications. In case of using low viscosity materials but larger nozzles, the silicone rubbers spread on the printing bed and dramatically impair minimal structure size and accuracy [11,12,15]. To overcome these limitations, processability of low-viscous materials is required.

Regarding the planned application field of sensing activities in neural tissue, medical grade silicone rubbers are necessary for long-term implants. Typically, most unrestricted medical grade silicone rubbers cure through temperature. Currently, no unrestricted UV-initiated silicone rubbers are available, which suggests inadequate qualification for medical approval and implantation for more than 28 days. To this end, temperature-vulcanizing materials for additively manufactured medical implants need to cure in reduced time, in a way that no delays between several minutes up to days exist during layer structuring. Luis et al. developed a novel heat-cured extrusion-based printer for silicone meniscus implants. They used heating elements on the nozzle and a heated printer bed for curing the silicone while printing the samples. Due to the used skin-safe certified silicone, an implantation of the printed meniscus implants is difficult to realize [16]. Another implementation for curing is presented in Stieghorst et al., where it was previously shown that employing an IR-laser for accelerated curing of silicone rubber droplets, a high reduction of spreading was achieved [17]. Porter et al. also used an IR-laser for curing medical grade silicone rubber but required fillers consisting of carbon black for suitable absorbance. Due to the fillers, however, the material is no longer unrestricted medical grade and thus not usable for implants, as planed in the current work [13]. Another approach is presented from Riahi et al. They used direct selective laser baking for 3D-printing of silicone rubbers. This idea is adapted from the conventionally used 3D-printing through stereolithography. The developed set-up is filled with an already mixed two-component silicone rubber and vulcanized layer by layer due to a CO_2_ laser at the desired positions. After each layer, the container is lowered, and a new layer of uncured silicone rubber is spread over the sample until the structure is complete build [18].

Conventional additive manufacturing technologies in which a laser is part of the printer set-up are, in addition to the already mentioned stereolithography, e.g., selective laser sintering or laser melting. For all approaches, the materials are filled in a container and exposed to the laser. After each layer, new material is covering the previous layer and exposure is repeated. Traditionally, stereolithography printers require photoactive materials, which are cured via an UV laser. Nevertheless, this technology is limited for unrestricted medical grade silicone rubber due to the already mentioned insufficient biocompatibility of the photoactive material. Looking at laser sintering or melting technology, powder-based materials are slightly or completely melted by use of a laser. Taking the paste-like texture of silicone rubber into account, it becomes clear that powder-based technology is not suitable for processing of silicone rubber [16,19].

Looking at mechanical properties of printed silicone rubber, it becomes apparent that some improvements are needed. If comparing conventional molded samples with 3D-printed silicone rubber samples, differences in tensile strength, tear strength, hardness, and elongation are reported from different groups [20,21]. For improvement, e.g., Jindal et al. optimized a room-temperature vulcanizing silicone rubber and tested 11 different formulations to reach the optimum combination of mechanical properties for 3D-printing of facial prostheses [15,22].

Next to the described cladding materials, conductive materials for electrodes of active implants are indispensable. Conventional neural implants are often manufactured in clean rooms using micro structuring processes [9,10]. For additive manufacturing of conductive materials, laser sintering is mainly employed [23]. Taking into account these manufacturing steps, it is not possible to combine the conventionally used extrusion techniques for silicone rubber and the laser sintering of conductive materials in a suitable common process for additively manufactured active implants. Additionally, patient-individual manufacturing and the desired flexibility of those materials are limited. As an alternative, conductive composites are becoming more popular. Generally, the composites are made from metal (e.g., silver, copper, gold) or carbon particles (e.g., carbon nanotubes, graphene) embedded in a matrix consisting of polymers or solvents [24]. Especially, polymer-based conductive materials are often used for improvement of mechanical properties, e.g., Nachtane et al. investigated carbon fiber-reinforced PolyEtherKetoneKetone (PEKK) composites in terms of their dynamic behavior with different amounts of fillers [25]. In addition, many groups improved, e.g., tensile strength or Young’s modulus with different fillers [19]. In addition to the mechanical improvement of materials, some groups focusing on the electrically properties of conductive materials for using these materials for flexible electronics [26,27,28]. Regarding medical applications, different composites are characterized, e.g., by Jakus et al., Yuk et al., and Kazemzadeh et al.. Jakus et al. printed first scaffolds for biomedical applications consisting of polylactide-co-glycolide and graphene. They characterized the mechanical and electrical properties, as well as the biocompatibility of the material [29]. Yuk et al. 3D printed a soft neural probe made from poly (3, 4-ethylenedioxythiophene): polystyrene sulfonate (PEDOT:PSS) and implanted it in mice for recording of neural signals [30]. Kazemzadeh et al. developed a bioink for commercial 3D bioprinters. Regarding the biocompatibility, in vitro and in vivo tests of the poly (glycerol-co-sebacate) (PGS)-based polymer and zinc composite showed promising results for flexible devices and implants. Furthermore, evaluation of conductivity, mechanical tests and rheological analyses were carried out [31]. 

To pave the way for individualized neural implants, a 3D-printing process capable of fabrication of biocompatible silicone rubber in combination with conductive pathways was developed. Fabricated linear electrode sensors using the process developed keep their biocompatible properties and allow for electrode impedances and diameters compatible with the specific requirements of neural implants.

## 2. Materials and Methods

### 2.1. Design and Components for the 3D-Printing Process 

To enable additive manufacturing of neural implants, a 3D-printing process tailored specifically to the requirements of the biocompatible materials selected were developed. The developed 3D-printing process employs an XY cross table with printing platform, a liquid dispenser for applying medical grade silicone rubber to the printing platform, and an IR-laser for accelerated vulcanization of the silicone rubber.

**Figure 1:**
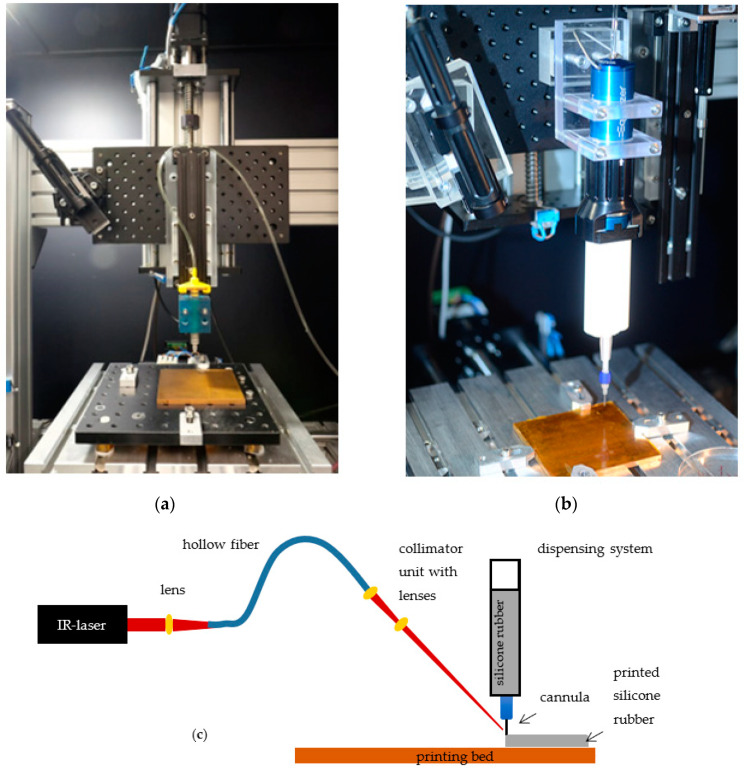
Design and components for the 3D-printing process: (**a**) One-component system, (**b**) two-component system; (**c**) schematic overview of 3D-printing process.

The XY cross table consists of two linear axes with blackflash-free recirculating ball nuts and step motors (PD4-C5918M4204-E-01, Nanotec, Feldkirchen, Germany), which are attached to the linear spindle with blackflash-free shaft coupling for increased resolution and repeatability. A copper printing bed is positioned on the XY cross table (Aluminum breadboard, Thorlabs, Newton, MA, USA), to which a polyimide film (Conrad Electronic, Hirschau, Germany) is attached. Polyimide films are used analog to results published previously [17]. Due to different materials properties, four heating elements are installed under the printing bed to be able to control the bed temperature in specified ranges. For thermal isolation of the axis, four blocks from polyetherimide are fixing the printing bed. Similarly, the Z-axis for adjusting the layer height is related with a linear axes and step motor (PD4-C5918M4204-E-01, Nanotec, Feldkirchen, Germany), as well, and is connected to a second platform (Aluminum breadboard, Thorlabs, Newton, MA, USA). The dispenser is fixated to the second platform and enables the application of desired material to the printing platform via compressed air (wall outlets). Depending on the materials properties and their recommended processing, the use of different systems is possible. Two dispensers are presented below (Figure 1). The Equalizer-2K-Dispenser (Nordson EFD, Feldkirchen, Germany) is used to process two-component (2K) materials. To this end, materials are filled into a double cartridge (50 cc, 7015724 Nordson EFD, Feldkirchen, Germany), placed in the dispenser and connected to the compressed air. To be able to apply material to the print bed, a static mixer (7701411 with Luer Adapter, Nordson EFD, Feldkirchen, Germany) and a nozzle (different inner diameter and designs depending on the application, Nordson EFD, Feldkirchen, Germany) are attached to the cartridge via a standard Luer-Lock connection. Through the dispenser, the volume flow can be adjusted by regulating the pressure. The second dispenser system Performus V (Nordson EFD, Feldkirchen, Germany) is recommended for one component (1K) materials. In this latter case a small single cartridge (3 cc, 7012074, Nordson EFD, Feldkirchen, Germany) is connected via Luer Lock to the nozzle. Due to the already mixed material, it is not necessary to use additional mixing components, such as the mixer. 

While the IR-laser (ULR-25, 9.3 µm, cw, 5 kHz Universal Laser Systems Inc., Vienna, Austria) is positioned next to the printing platform, a collimation unit is fixed on the platform of the Z-axis to achieve a constant alignment of the laser beam on the nozzle tip. To be able to use the laser beam for vulcanization, the laser radiation must be transmitted to the collimation unit via a flexible hollow core fiber (Hollow Silica Waveguide, CO_2_ laser optimized, Acrylate, Laser Components, Olching, Germany). This requires three zinc selenide plano-convex lenses. After the beam has been emitted from the laser, a lens (LA7477-G, Thorlabs, Newton, MA, USA) is integrated before coupling into the hollow core fiber. Two additional lenses (LA7477-G, LA7542-G, and LA7753-G, and Lens Tube SM05L30C, Thorlabs, Newton, MA, USA) are placed after the hollow core fiber in the collimation unit that allow the adjustment of the beam diameter. For alignment of the laser, an IR-detection card (MIR liquid crystal detector card VRC6, Thorlabs, Newton, MA, USA) and a goniometer (GNL10/m, Thorlabs, Newton, MA, USA) are used. Since the IR-Laser employed in the process belongs to class 4 (DIN EN 60825-2), protective measures are necessary. To this end, the printing setup is positioned in a protective housing made from aluminum profiles (alcom international GmbH, Reutlingen, Germany) and metal sheets (Stahljunge GmbH, Berlin, Germany), which are equipped with a black diffuse surface coating to reduce reflections. Safety switches (SKI-U1Z M3, Bernstein AG, Porta Westfalica, Germany), in addition, ensure that the laser power source is switched off upon opening of the housing. An emergency stop and warning notices on the printer, according to DIN EN 60825-1, are also required. To still be able to follow the printing process inside of the setup, a radiation-proof window (Laservision GmbH, Fürth, Germany) was integrated in the housing. Overall, the implementation of the housing makes it possible to reduce the laser class from class 4 to class 1, which means that there are no risks for the user when used as intended, and no special protective measures (such as laser safety goggles) are necessary.

The step motors of the printer axes are controlled by a microcontroller (Arduino uno). In combination with an opensource GRBL G-code interpreter (Universal G-Code-Sender), it is possible to print the desired samples according to G-Code files.

### 2.2. Sample Fabrication

To demonstrate applicability of the developed printing process, various samples have been produced. They all consist of platinum-catalyzed addition-curing silicone rubber Silpuran 2430 (RTV-2, Wacker Chemie AG, Burghausen, Germany) and electrically conductive, silver-containing epoxy resin EpoTek H20E (Epoxy Technology Inc., Billerica, MA, USA). In order to be able to produce a neural implant, an assessment of the biocompatibility after the curing process with IR-laser is essential. Furthermore, with regard to electrical properties, a classification of the samples is necessary. Thus, biocompatibility assessment and impedance measurements were performed.

- Sample fabrication for biocompatibility assessment

To assess the biocompatibility of the silicone rubber after printing the material and curing by laser radiation, samples were fabricated and tested for cytotoxicity as a key aspect of biocompatibility of materials in line with EN ISO 10993-5 (2009) [32]. Similarly, cytotoxicity of the conductive epoxy resin was investigated. To take possible irregularities during production into account, the samples were produced in 3 batches of 6 samples each.

*Silicone rubber samples:* For the silicone rubber samples, approximately 3 × 3 cm large areas were printed through the 3D-printing process developed. The double cartridge system with spiral mixer, nozzle, and electromechanical dispenser as described above was used. A pressure of 5.7 bar and a duty cycle of 6 (0.69 W/mm^2^) with a feed rate of 500 mm/min enabled the desired sample sizes. To ensure complete curing and removal of volatiles after the printing process, tempering on the heating plate at 150 °C for 15 min were performed [33]. To compare the printed samples with conventionally vulcanized silicone rubber samples, an almost identical fabrication was carried out with the 3D-printing process, but without laser radiation. In this case, the vulcanization of the silicone rubber took place only on the heating plate. All printed samples were cut into 4 × 4 mm pieces and autoclaved at 121 °C.

*Conductive epoxy resin:* Due to existing solvents in the conductive polymer and the evaporation of those, the laser was not used to cure the epoxy resin. For this reason, the samples were produced in the conventional way, as described in the data sheet. In brief, samples were knife-coated on polypropylene films at a thickness of 0.3 mm and vulcanized at 150 °C for 1 h. To enable the fabrication of the sample sizes of 4 × 4 mm, the size was already defined before vulcanization. After curing, the samples were autoclaved.

Eighteen samples are taken into account for the EpoTek and the Silpuran 2430 and 6 samples each for the conventionally produced silicone rubber samples and the negative controls.

- Sample fabrication for impedance testing

**Figure 2:**
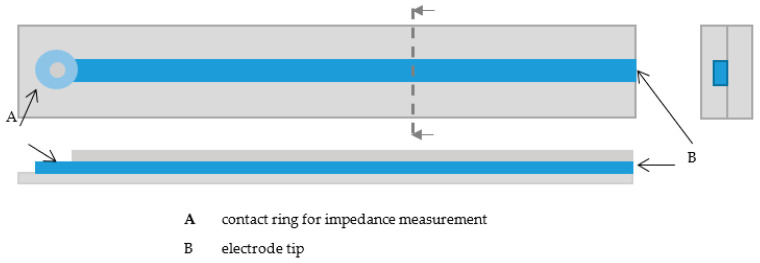
Schematic layout of samples for impedance measurements and determining the structure size.

The samples for impedance measurements were printed with the developed 3D-printing process. All samples were 4-cm long and consisted of two silicone rubber layers and the conductive epoxy resin. To be able to perform the impedance measurements, a small ring was printed at one end of the line, on which the contact to the impedance measurement device could be made (Figure 2). To determine effects of the line thickness and height on the impedances, linear electrodes with different printing parameters were printed. To determine the reached size, line widths and heights were measured separately with a microscope (Stemi-2000-C, Zeiss, Oberkochen, Germany). Therefore, line widths and heights were measured at 12 electrodes at different positions (*n* = 4 per electrode).

The first silicone rubber layer was printed using the electromechanical 2K dispenser (Equalizer, Nordson EFD, Feldkirchen, Germany), a double cartridge (50 cc, 7015724, Nordson EFD, Feldkirchen, Germany), a spiral mixer (7701411 with Luer Adapter, Nordson EFD, Feldkirchen, Germany), and a 250 µm nozzle (7018333, Nordson EFD, Feldkirchen, Germany). To obtain the desired samples, a pressure of 5.7 bar and a duty cycle between 6–8 (0.69–1.08 W/mm^2^) were set. The duty cycle was reduced at the end of the printing process as the continuous laser radiation lead to heat development (and thus to degradation of the material). The feed rate was 300 mm/min during the printing process. After printing, the samples were tempered on a heating plate to ensure complete vulcanization (150 °C, 15 min). In a next step, the conductive layer was printed with the dispenser Performus V (Nordson EFD, Feldkirchen, Germany). Due to the required curing temperature of at least 80 °C, the material could be mixed before starting the printing process. Consequently, there was no need for a mixer, and only a small cartridge (7012074, Nordson EFD, Feldkirchen, Germany) with nozzle (Ø 0.15 mm and Ø 0.2 mm, 7018462 and 7018395, Nordson EFD, Feldkirchen, Germany) could be used. The conductive epoxy resin was applied at a pressure of 1.9 bar and 2.5 bar, while the feed rate is 300 mm/min on the straight sections and 200 mm/min in the curves. Due to variation of parameters, it was possible to produce lines with different widths and heights. The vulcanization process took place on the heating plate at 150 °C for 5 min, in accordance with the data sheet. To complete the samples, another layer of silicone rubber was printed. This was done similarly to the first layer. Differences arise in the feed rate (100 mm/min) and in a reduced duty cycle up to zero, depending on the heat development. An overview of printing parameters for the linear electrodes is shown in Table 1.

### 2.3. Biocompatibility Assessment

To enable application of future medical devices built employing the developed printing process, the applicable standards towards regulatory approval should be considered. For materials in direct contact with patients, a decisive standard is the biological evaluation of medical devices according to EN ISO 10993. There, the testing for cytotoxicity (ISO 10993-5) is a common method to assess in a first approximation whether a material is biocompatible or not. Hence, in this work, cytotoxicity of silicone rubber and for a conductive epoxy with silver particles for a neural implant were evaluated. Despite starting with a material described as biocompatible, for the silicone rubber, potential negative effects due to the laser radiation during the vulcanization process had to be excluded to ensure applicability in human patients. For this purpose, the samples cross-linked with the laser were compared with conventionally produced samples. For the conductive material, the suitability for neural implants should be evaluated.

To investigate the metabolic activity of native murine NIH-3T3 fibroblasts, a water-soluble tetrazolium dye (WST-1, Cell proliferation Reagent WST-1, Roche GmbH, Basel, Switzerland) was used for testing the in vitro cytotoxicity of samples in line with ISO 10993-5.

Cells (lentivirally GFP-labeled murine NIH3T3 fibroblast) were cultured in three different culture flask with Dulbecco’s modified Eagle’s medium (DMEM, Biochrom, Berlin, Germany) (without phenol red) supplemented with 10% fetal calf serum (FCS, Biochrom, Berlin, Germany) and 0.5% L-Glutamin (Biochrom, Berlin, Germany)

When the flasks were 85% full, the cells were trypsinized and counted. The silicone rubber and conductive material samples were placed into each well of a 96-well polystyrene microplate. Dimethyl sulfoxide (DMSO, Carl Roth, Karlsruhe, Germany) was added as a positive control, with DMEM as a negative control. Subsequently, 10,000 fibroblasts were pipetted onto the sample in each well and incubated for 48 h at 37 °C and 5% CO_2_. Next, 10 µL of WST-1 solution was added to each well and incubated again for 30 min (37 °C, 5% CO_2_). Due to sample color, it was necessary to pipette 100 µL from each well into a new well plate. Finally, the optical density was measured via a microplate photometer (Synergy H1 Multi-Detection-Reader, BioTek Instruments, Vermont, VT, USA) using a 450 nm filter following the manufacturer’s protocol. The calculation of the cell viability was performed according to equation 1, where the cell viability in percent (Cell viab.%) is calculated from the mean optical density of a certain sample (OD_450s_) and the mean optical density of the control wells (neg. control, cell grown on untreated wells) (OD_450c_). The viability of the cells in a well without samples was considered 100% cell viability and used for comparison.

Cell viab.% = 100 × OD_450s_/OD_450c_. (1)

### 2.4. Impedance Measurements

To assess the suitability of the conductive material as an electrode material, impedance measurements were carried out. Depending on the planned application, an implant requires different minimal structure sizes. Hence, linear electrodes with different parameter settings between two cladding silicone rubber layers were printed. For the two types of electrodes, a mean value for line widths and heights and for the measured impedances were calculated. Linear electrodes with smaller structure size were printed with a smaller nozzle and a low dispensing pressure. Electrodes for implants where an adequate structure size is sufficient were printed with a larger nozzle and a higher dispensing pressure (for detailed description cf. Section 2.2).

Impedance measurements (IM6, Zahner-Elektrik GMBH, Kronach, Germany) were performed by applying 10 mV DC at 1 kHz. A platinum counter electrode was used in physiological saline solution (NaCl 0.9%, B. Braun, Melsungen, Germany). The electrodes were contacted through the middle of the ring, and the impedance between the end of the electrode and the counter electrode were measured. Furthermore, impedance measurements at different frequencies (1–100 kHz) were carried out. Therefore, a three-electrode system was used with the printed electrode as the working electrode, a platinum counter electrode, and Ag/AgCl as reference electrode in physiological saline solution.

### 2.5. Data Analysis

Data obtained through the printing process, biocompatibility assessment, and impedance measurements were collected and stored using Excel. Data analysis was performed in Excel and/or MATLAB (MathWorks, Natick, MA, USA) using implemented routines.

## 3. Results

### 3.1. 3D-Printing Setup

After all components had been successfully assembled for the 3D-printing process, the setup could be used for fabrication. Due to the straight forward design of the setup, the components can be easily removed or changed, which, for example, enables usage of different dispensers. Because of a large platform of the Z-axis, two dispenser systems (1 K and 2 K) can be used in parallel if the laser is only used for one material. For laser spot alignment and prevention of clogging of the nozzle due to increased temperature, a goniometer and the IR-card is used. Due to the fixation of the laser and dispenser on the same platform, a perfect alignment in a printing process is guaranteed.

**Figure 3:**
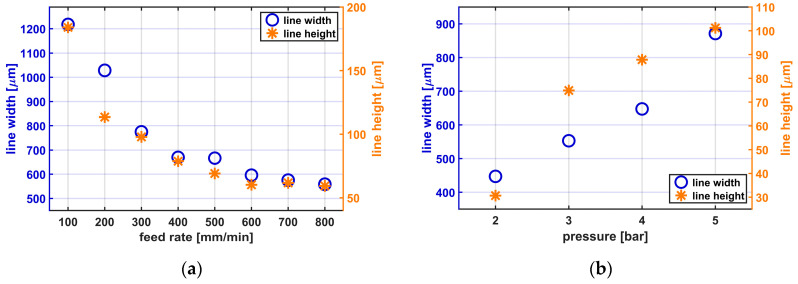
Dependence of line width and height on feed rate and pressure with Silpuran 2430. (**a**) Average line widths and heights for different feed rates: 0.25 mm nozzle, 5.7 bar, duty cycle of 8 (*n* = 6); (**b**) average line widths and heights for different printing pressures: 0.25 nozzle, feed rate of 300 mm/min, duty cycle of 8 (*n* = 6).

While several changes of process parameters and their consequences are known well, only two of the main effects of adjusting the printing structure size are shown. Figure 3a,b shows the consequences of varying the feed rate and the printing pressure, respectively, while printing Silpuran 2430.

When the feed rate is increased, the line widths and heights decrease. With a 0.25 mm nozzle, applying a constant pressure of 5.7 bar, a duty cycle of 8, and the varying feed rate, line widths between 558.8 ± 22.6 µm (mean ± standard deviation) and 1218.2 ± 41.5 µm are achieved. The line height is also influenced by the feed rate and varies between 59.4 ± 1.3 µm and 183.9 ± 16 µm. When considering the influence of the printing pressure on the structure size, it becomes clear that with lower pressure and thus volume flow, narrower, and flatter lines are reached. With a 0.25 mm nozzle, a constant feed rate of 300 mm/min, a duty cycle of 8, and the varying applied pressures between 2 and 5 bar, line widths between 447 ± 10.7 and 871.2 ± 3.2 µm are achieved. The line height varies depending on the volume flow between 30.6 ± 7.4 and 101.2 ± 7.8 µm. All presented results are mean values from 24 measuring points.

It also became clear that the duty cycle needs to be adapted depending on the feed rate and the volume flow. If the duty cycle is too high, clogging of nozzle, or, in the worst case, thermal degradation of the silicone rubber can be observed. In addition, before starting the printing process, it is necessary to control the distance between nozzle and printing bed. Depending on the used parameter, different minimal structure sizes can be detected.

Regarding mechanical properties of printed samples, tests according to ASTM D-3163 were carried out. Based on this standard, the bonding strength between two silicone rubber layers was investigated. Tests with a measurement velocity of 50 mm/min showed no delimitation between the layers and results in reliable bonding strength for printed silicone rubber samples.

### 3.2. Biocompatibility Tests

**Figure 4:**
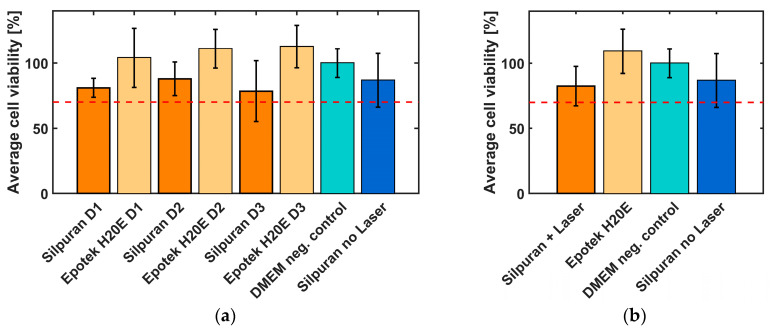
Biocompatibility of Silpuran 2430 and EpoTek H20E. (**a**) Cell viability of Silpuran 2430 (vulcanization with IR-laser and conventional) and EpoTek at three different days (each *n* = 6, mean ± standard deviation); (**b**) summary of all measured cell viabilities (*n* = 18, mean ± standard deviation for Silpuran 2430 and EpoTek and n = 6 for negative control and conventionally vulcanized Silpuran 2430).

A WST1 test in line with ISO 10993-5 was carried out to investigate and evaluate the effects of laser radiation on the cytotoxicity of the silicone rubber Silpuran 2430. To be able to compare the biocompatibility of the 3D-printed samples, conventionally vulcanized silicone rubber samples were also fabricated. In addition to the silicone rubber, the biocompatibility of the conductive material EpoTek H20E of the electrode contacts and wires was investigated to assess the suitability of the material for implants. The samples were produced on different days and also tested with different cell suspensions to exclude external influences (Figure 4a, *n* = 6 each). The silver epoxy samples reached a cell viability of >100%. In comparison, the biocompatibility of the silicone rubber was lower. However, it should be noted that only minor differences can be identified between the conventionally and laser vulcanized samples. The standard deviation of all samples was found to be between 7.3% and 23.3%.

The summary of all results is shown in Figure 4b. All samples show a cell viability of more than 70%, which indicate non-cytotoxic behavior of the tested materials.

### 3.3. Impedance Measurements

**Figure 5:**
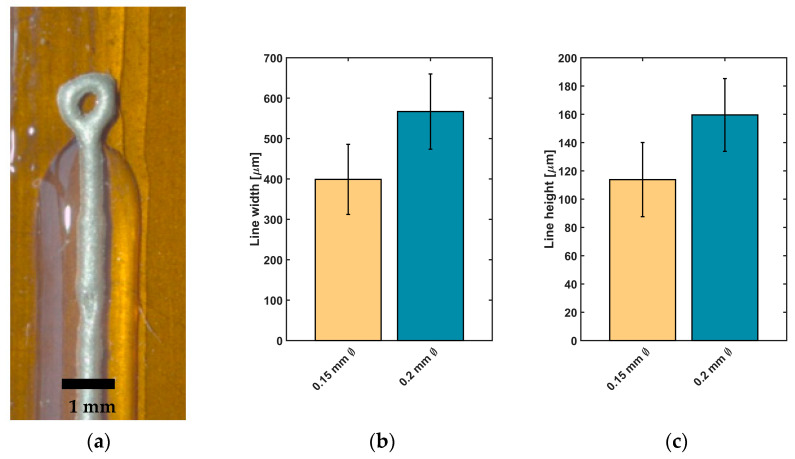

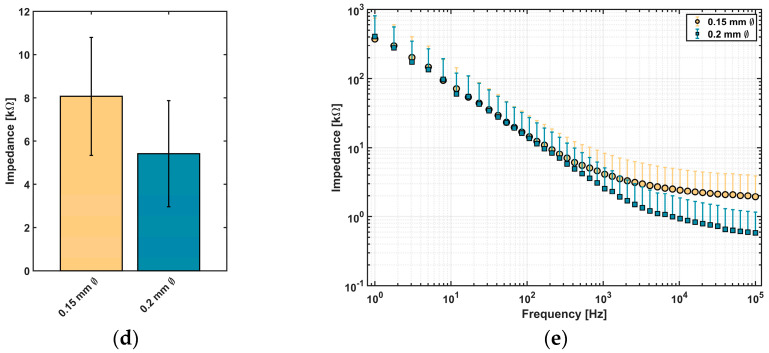
Line widths, heights and measured impedances of printed linear electrodes consisting of Silpuran 2430 and EpoTek H20E. (**a**) printed linear electrodes, black bar = 1 mm; (**b**) line widths of linear electrodes printed with different nozzles and printing parameters (*n* = 24, mean ± standard deviation); (**c**) line heights of linear electrodes printed with different nozzles and printing parameters (*n* = 24); (**d**) measured impedances (1 kHz) for linear electrodes printed with different nozzles and printing parameters (*n* = 14, *n* = 16); (**e**) impedance spectroscopy (1–100 kHz) for linear electrodes printed with different nozzles and printing parameters (only the positive standard deviations are depicted for improved visibility).

Impedance measurements were carried out to assess the suitability of conductive epoxy for neural implants. Depending on desired structures of the implant, linear electrodes with two different structure sizes were printed between two cladding silicone rubber layers (Figure 5a). For the two types of linear electrodes, a mean value for line width and height and for the measured impedance was calculated and is presented in Figure 5b–e).

Electrodes printed with Ø 0.2 mm nozzle reached a line width of 566.8 ± 93.1 µm and height of 159.6 ± 25.7 µm (*n* = 24 each). For electrodes printed with a smaller nozzle, a reduction of line width to 398.9 ± 86.7 µm and a decrease of line height to 113.8 ± 26.1 µm were achieved (*n* = 24 each).

Regarding the impedance values of printed electrodes, Figure 5d visualizes that a reduction of line width leads to an increase in impedance. For lines with a higher structure size, a value of 5.4 ± 2.5 kΩ (*n* = 16) at 1 kHz was measured. In comparison to lines with smaller structure sizes, an impedance of 8.1 ± 2.7 kΩ (*n* = 14) at 1 kHz was detected. Spectroscopic measurements of impedance over a frequency range of 1–100 kHz are showing comparable results (Figure 5e): The measured impedance of lines printed with the smaller nozzle (Ø 0.15 mm) tends to higher impedance values than the values of electrodes printed with the Ø 0.20 mm nozzle, especially at higher frequencies in the kHz-range.

**Figure 6:**
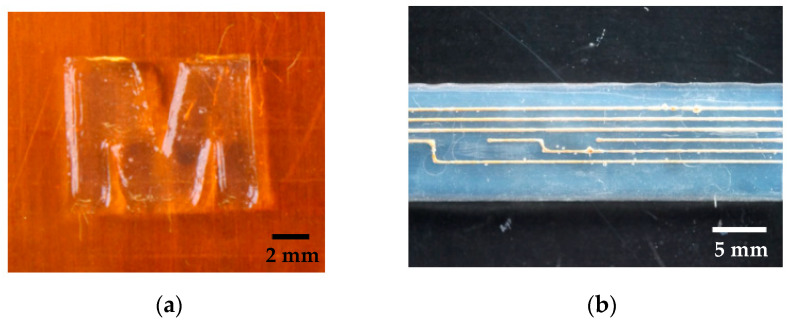
Different samples consisting of medical grade silicone rubber. ((**a**), bar = 2 mm) and consisting of silicone rubber with conductive materials ((**b**), bar = 5 mm) display the versatility of the developed 3D-printing process.

In summary, the developed manufacturing process allows printing of different structures consisting of different materials. Depending on the desired design, a variety of samples can be manufactured. Figure 6 shows two different samples as an example. 

## 4. Discussion

### 4.1. 3D-Printing Process Development

A 3D-printing process for the manufacturing of medical grade silicone rubber implants was developed and assessed by means of first linear electrode test samples. While 3D-printers for silicone rubber are already available on the market, their use in the medical field is limited. There are two main approaches of printing silicone rubbers. On one hand, the vulcanization takes place in layers by UV radiation (e.g., ACEO, Burghausen, Germany, or Envisiontec, Gladbeck, Germany) and, on the other hand, by vulcanizing the whole samples after the printing process (e.g., Envisiontec, Gladbeck, Germany, or German RepRap GmbH, Feldkirchen, Germany). Depending on the material, the curing take place at room temperature or at higher temperatures for several minutes up to days.

It is often reported that the usage of viscous silicone rubber is recommended so that is does not spread and ensure high shape retention capability, high flexibility, and resilience [11,12,15]. By comparing temperature- and UV-curing silicone rubbers, Liravi et al. showed that the printing of UV-curing silicone rubbers will be a further growing field in the next years [12]. The reason for that is the more practicable curing process directly after printing of the layer.

For the long-term implantable devices envisaged here, medically approved materials for a period of more than 28 days are essential. However, there are only a small number of unrestricted silicone rubbers and none of them cure by UV radiation. For this reason, it can be assumed that the UV-curing contradicts unlimited medical approval. Unrestricted approved silicone rubbers (e.g., Nusil from Avantor) are often cures with heat via addition-cure chemistry, with platinum addition cure or require the addition of a peroxide catalyst to accomplish the vulcanization.

To achieve the smallest possible structure size for complex geometries, nozzles with the smallest possible inner diameter (<100 µm) are essential. However, especially when using 2 K systems, higher pressures are required to apply material and suitable mixing of the components at low volume flows need to be taken into account. In addition, the sometimes very high viscosities of the materials limit the use of nozzles with small inner diameters. If, on the other hand, materials with lower viscosities are used, noticeable higher structure sizes are achieved because the material spreads out to a maximum on the printing bed when it flows out of the nozzle. As a consequence, it cannot be printed in a dimensionally stable design [12,15].

All these challenges can be overcome with the developed printing process. Due to the immediate, punctiform vulcanization of the materials directly after flowing out of the nozzle, the spread of low-viscosity materials is limited and allows immediate applying of the next layer in the desired structure size. Due to the pronounced absorption bands of silicone rubber (polydimethylsiloxane) in the infrared wavelength range, an IR-laser has proven to be particularly suitable for an immediate increase in temperature and enables the production of various test samples [17]. The processing time for vulcanization with IR is even shorter compared to curing materials with UV. At the end, the developed printer with IR curing represents a feasible alternative for time saving processing of polymeric temperature-curing materials in patient individual designs.

There are different groups who are using IR-lasers for curing of silicone rubber. Porter et al. used an IR-laser (1064 nm) for curing medical grade silicone rubber. Because of degradation of the materials before crosslinking they used carbon filler for increasing the IR absorbance for reaching printing results [13]. Regarding to the filler employed, it becomes clear that the filler prevents the unrestricted use for long term implants. In the developed printing process, it is not necessary to mix in fillers for better absorbance because, as shown in previous work [33], the highest absorption bands for silicone rubbers is in the wavelength range of 9.3 µm. Using a suitable laser with the desired wavelength thus allows for accelerated curing of the silicone rubber. Regarding the heat development while printing, the same necessity of reducing the laser power during the manufacturing process was found. Another approach to vulcanization with an IR-laser is described by Riahi et al. In this work, a liquid printing bed filled with silicone rubber is used. For printing structures, the laser is focused in the liquid silicone rubber and vulcanizes the material in desired areas. At the end, excess material is removed. For products only consisting of silicone rubber, it seems to be an alternative, but, in case of printing sensors, it will not be possible to print conductive materials between silicone layers in the same printing set-up [18]. Due to limited penetration depth of IR radiation into the material, it can be assumed that mainly the surface is covered with vulcanized silicone rubber. According to finite element method simulations of Zhang et al., high internal stress arises in materials as the layer height increases. Accordingly, low volumes should preferably be printed in order to reduce high changes in temperature in one layer [18]. Despite the low volumes that should be printed, the developed printing process is suitable for medical applications. In most cases, small structure sizes are necessary; therefore, as shown in Figure 3, low volumes must be applied.

To correctly use the developed 3D-printing process, separate safety precautions are necessary. Due to the integration of the IR-laser in the printing set up and the resulting electromagnetic radiation, a housing and several safety levels, as well as instructions, are needed. The option to integrate different dispensers in the set-up allows the use of different materials. Depending on the dispenser, one-component or two-component systems can be implemented. The 2 K dispenser is also designed for different mixing ratios and can expand the range of applications. Furthermore, the dispensers allow a material-specific setting of the volume flow due to the adjusted air pressure (between 0.1 and 6 bar). A low pressure applies less material and leads to narrow and flatter lines. In contrast to that, using higher pressure produces higher and wider lines due to the larger volume which is applied in the same time. Due to the use of Luer Lock cartridges, individual nozzles (Ø 0.1–1.54 mm) can be selected for each printing process and directly influence the printed structure size. The software of the printer enables to set the feed rate between 100 and 1000 mm/min and thus also to adjust the printed structure size. A high feed rate leads to a decrease in line width and line height. While the setting of the structure size through the printing pressure, the feed rate, the distance between the printing bed and the nozzle, and the nozzle diameter is already known [12,14], the laser power also plays a decisive role in this work [17]. When choosing a slow feed rate, a low laser power, and volume flow, higher structure sizes are achieved. On the other hand, higher feed rates, lower volume flows, and high laser powers lead to smaller structure sizes.

Generally, the viscosity of the silicone and the used nozzle mostly influence the structure size reached. While many published results show structure sizes larger than 0.2 mm, some groups are able to reach minimal structure size up to 10 µm [14]. Since the printing time increases with the structure size, it is essential to weigh up between printing time and structure size, depending on the application.

First mechanical characterization of printed samples in accordance to ASTM D 3163 resulted in reliable bonding strength and showed no delamination between different silicone rubber layers.

Due to the set-up of the printer it is possible to use different liquid and paste-like materials at the same time. The integrated IR laser allows accelerated curing of silicone rubbers and enables direct processing of the next layer. Due to the IR laser, spreading of low viscous materials is inhibited. By processing conductive materials, medical devices as active neural implants or other sensing devices can be manufactured.

### 4.2. Biocompatibility

According to ISO 10993-5 (in vitro cytotoxicity), a cell viability percentage of more than 70% indicates non-toxic properties of the sample. Since it is not guaranteed that the mean optical density of the control wells is higher than that of the sample wells, it is possible to achieve cell viability values of >100%. In this case, the cells thrived better on the samples, than on the control surface (DMEM). This mainly occurred with the EpoTek H20E samples. In some cases, these are above 100% cell viability. In comparison, the biocompatibility of the silicone rubber is lower. However, it is noticeable that only slight differences can be seen between the conventionally and laser-vulcanized samples. Overall, it can be said that all cell viability is above 70% and thus indicates a material with biocompatible properties [32].

According to the manufacturer, Silpuran has been successfully tested in accordance with DIN EN ISO 10993-5, which has also been confirmed in this work. In addition, it could be shown that the IR radiation does not negatively affect the biocompatibility of the silicone rubber and that the IR-laser seems suitable for the additive manufacturing of medical grade silicone rubber implants. Residual products, such as un-crosslinked linear polydimethylsiloxane chains or residual crosslinking agent, which could remain in the silicone rubber due to insufficient vulcanization would have a negative effect on biocompatibility. As expected, this cannot be seen from the results and can be avoided by the post-curing that follows each printing process [33,34].

When comparing the cell viability of the two materials tested, it is noticeable that the cells apparently prefer to grow on the conductive material. Here, cell viability levels of over 100% are measured, while the viability for silicone rubber is between 79 and 88%. These results are promising for the planned application in which the conductive material acts as an intimate cell-electrode interface, depending on the application possibly even facilitating neurite outgrowth. This could lead to optimized diagnosis or treatment results and seems to be a promising approach in comparison to conventionally produced electrodes.

Although the results from cytotoxicity testing are considered a strong predictive measure for a successful long-term application of medical devices, they do not inform about the continuous function in the long-term chronic application. Especially, in applications involving electrical sensing of neural activity, the long-term functions are often impaired through insulation effects resulting from tissue response. Consequently, as next steps, the tissue response after insertion or implantation into brain tissue should be investigated in terms of blood-brain-barrier breach, astrocytic response, markers for activated macrophages, and the presence of microglia [3,4].

### 4.3. Impedance Measurements

Three dimensional-printing of conductive materials is currently limited to powder-based materials, which are solidified in different ways (e.g., selective laser sintering [23]). Regarding the designed set-up of the 3D-printer, it is not possible to handle powder next to the liquid silicone rubber. A second point of view is the flexibility of those materials, which need to be much higher [5,7,8]. As an alternative, a conductive polymer-based material in combination with flexible silicone rubber were investigated. For first classification, linear electrodes were printed with different structure sizes to categorize the measured impedance values. Regarding the structure size of the printed conductive samples, mainly the particle sizes of the conductive filler are determining the minimal structure size. Depending on the relation between nozzle diameter and particle size, e.g., loss in conductivity or agglomeration are possible and limit the functionality of the electrodes. To achieve lines smaller than 0.4 mm, conductive fillers with particles sizes in one-digit (to two-digit) nm-scale should be used to be able to take into account the factor 50 between the particle size and nozzle inner diameter [35]. Depending on the implant location, materials with different particle sizes are needed for more precise electrodes and as a result more precise interfacing with neural tissue.

Electrode impedance is the important measure for application as sensors of electrical neural activity [36]. There, higher values ± 0.9 MOhm are generally suitable for high spatial and temporal resolution recording of single cell action potential activities, while lower values in the kOhm (≤50 kOhm) range are applicable for lower resolution recording of oscillations from cell networks [6]. Since a first goal of the developed 3D-printing process is to enable manufacturing of individualized medical devices for improved diagnosis, e.g., in temporal lobe epilepsy, electrode diameters and impedances comparable to devices used for electrocortical grid recordings in epilepsy diagnosis and treatment (e.g., Ad-Tech Medical Instrument Corporation, Racine, WI, USA, PMT Corporation, Chanhassen, MN, USA, Inomed Medizintechnik GmbH, Emmendingen, Germany) were targeted. With the resulting single-digit kOhm impedances of the fabricated electrodes and recording surface in the < mm range, the printed devices fall exactly into that range useful for assessment of oscillatory epileptiform activities.

Reynold et al. investigated the viability of conductive medical epoxy as an implantable electrode material. They fabricated different electrodes with the same material as in this work and concluded, in agreement with this work, that the potential of conductive medical epoxy for biopotential recording electrodes can be indicated by first electrical assessment of the interface [37]. In addition, the printed linear electrodes are comparable to conventional clean room electrodes [9]. Rubehn et al. designed an electrode array with 252 electrodes on 14 shafts with an electrode diameter of 1 mm. There, the impedance values varied from 1.5 kΩ to 5 kΩ (measured at 1 kHz DC), similar to the presented results. Comparing the impedance over a frequency range yielded similar results [9]. Furthermore, Lu et al. showed similar results performing impedance spectroscopy with nanowire-coated fibers for optoelectronic probing of spinal circuits, especially at 1 kHz [38].

In addition, regarding the planned application, coatings can be an option for surface modification to tune impedance characteristics of the electrodes, as already known from ECoG micro arrays and cochlear implant electrode arrays [10,39].

## 5. Conclusions

The developed 3D-printing process can enable patient-individual printing of biocompatible, flexible, active implants and is a promising approach for optimized diagnosis and treatment of different diseases. Due to the integrated IR-laser, accelerated curing of temperature-initiated medical grade silicone rubber allows faster structuring of layers of low-viscosity silicone rubber with reduced spreading. Depending on the used printing parameters (e.g., printing pressure, duty cycle, feed rate, and nozzle diameter), adjustment of the desired structure size is possible. Results of cytotoxicity tests showed no influence of laser-cured samples on biocompatibility in comparison to conventionally cured samples. Although the material used here, Silpuran 2430, is a restricted approved medical grade silicone rubber, there are no reservations in combination with the IR-laser for unrestricted medical grade silicone rubbers, since the vulcanization process is also temperature driven. Furthermore, in combination with conductive polymer-based materials, first linear electrodes with the developed printing process were printed. Regarding conventional manufacturing of electrode arrays, this process offers patient-individual manufacturing of the whole device in one process. Characterization of the electrical properties by means of impedance measurements shows results in a range of conventional sensors for the recording of neural activity and thus fulfills the requirements for this application field. Additionally, biocompatibility tests of the conductive material showed increased affinity of cell on the polymer-based silver epoxy as for the silicone rubber, which could lead to improved diagnosis and treatment due to an improved interface between device and cells.

In summary, a 3D-printing process for the manufacturing of patient-individual neural implants was developed. Due to the integrated IR-laser, accelerated thermal curing of unrestricted medical grade silicone rubber allows structuring of lower viscous materials. In combination with polymer-based conductive materials, first, sensors were printed in consecutive fabrication steps and showed promising results regarding their biocompatibility and electrical properties.

## Figures and Tables

**Table 1:** Printing parameters for linear electrodes.

Layer	Material -	Feed Rate mm/min	Duty Cycle -	Pressure Bar	Nozzle Ø mm	Dispenser -
1. layer	Silpuran 2430	300	6–8	5.7	0.25	2 K
2. layer	H20E	300 electrode tip 200 contact ring	-	1.9 2.5	0.15 0.2	1 K
3. layer	Silpuran 2430	100	0–8	5.7	0.25	2 K

## Abbreviations

IR	infrared
ECoG	electrocorticography
UV	ultraviolet
3D	three-dimensional
1K	one component
2K	two component
RTV2	room temperature vulcanizing silicone rubber consisting from 2 components
WST1-test	water-soluble tetrazolium dye
